# Monolithic GaN-Based Dual-Quantum-Well LEDs with Size-Controlled Color-Tunable White-Light Emission

**DOI:** 10.3390/ma18092140

**Published:** 2025-05-06

**Authors:** Seung Hun Lee, Dabin Jeon, Gun-Woo Lee, Sung-Nam Lee

**Affiliations:** 1 Department of IT Semiconductor Convergence Engineering, Tech University of Korea, Siheung 15073, Republic of Korea; 2 Department of Semiconductor Engineering, Tech University of Korea, Siheung 15073, Republic of Korea

**Keywords:** GaN, quantum well, white LED, band-filling effect, color temperature

## Abstract

We report a monolithic GaN-based light-emitting diode (LED) platform capable of color-tunable white-light emission via LED size scaling. By varying the LED size from 800 µm to 50 µm, the injection current density was effectively controlled under constant driving current, enabling precise modulation of carrier distribution within a dual-composition multi-quantum well (MQW) structure. The active layer consists of five lower In_0.15_Ga_0.85_N/GaN QWs for blue emission and strain induction, and an upper In_0.3_Ga_0.7_N/GaN single QW engineered for red-orange emission. The strain imposed by lower QWs promotes indium segregation in the last QW through spinodal decomposition, resulting in a broadened emission spanning from ~500 nm to 580 nm. High-resolution TEM and EDX analyses directly confirmed the indium segregation and phase-separated structure of the last QW. Spectral analysis revealed that larger devices exhibited dominant emission at 580 nm with a correlated color temperature (CCT) of 2536 K and a CIE coordinate of (0.501, 0.490). As LED size decreased, increased hole injection allowed recombination to occur in deeper QWs, resulting in a blueshift to 450 nm and a CCT of 9425 K with CIE (0.224, 0.218) in the 50 × 50 µm^2^ LED. This approach enables phosphor-free white-light generation with tunable color temperatures and chromaticities using a single wafer, offering a promising strategy for compact, adaptive solid-state lighting applications.

## 1. Introduction

White-light generation in GaN-based light-emitting diodes (LEDs) is a critical area of research in solid-state lighting and display technologies [1,2,3]. The ability to engineer white emission directly from compact and energy-efficient sources has enabled advances in applications ranging from general illumination to full-color displays [4,5,6]. Traditional white LED technologies typically employ blue LEDs in combination with yellow phosphor coatings, most notably YAG phosphor, to produce broadband white light [5,6,7]. Although widely adopted, this approach suffers from several drawbacks, including color instability over time, poor color rendering index (CRI), and limitations in high-temperature performance [8,9]. To overcome these issues, researchers have explored alternative strategies for phosphor-free white-light generation [10,11,12,13,14]. One such method involves integrating multiple color emitters—such as red, green, and blue (RGB) chips—within a single package to achieve white emission via additive color mixing [3,4]. However, this method requires complex electrical driving circuits and suffers from spatial color separation [3]. Recently, nanostructured and emerging materials such as colloidal quantum dots (QDs), perovskites, and nanowires have attracted significant attention owing to their wavelength tunability and high photoluminescence quantum yields [7,15,16,17]. In particular, perovskite-based LEDs (PeLEDs) show promise for white-light generation owing to their solution processability and spectral versatility. Nonetheless, stability under electrical bias and environmental exposure remains a key limitation of these materials [7]. Similarly, quantum dot LEDs (QLEDs) offer high color purity and tunable emission; however, they still face challenges related to long-term operational stability. In contrast, GaN-based LEDs provide superior chemical and thermal robustness, making them more suitable for high-power and reliable solid-state lighting applications.

Among these approaches, achieving intrinsic monolithic white emission through engineered quantum well (QW) structures in III-nitride semiconductors is a promising and stable solution [10,11,12]. By tailoring the thickness and indium composition of each well in a multi-quantum well (MQW) structure, multiple emission wavelengths, from blue to red, can be generated simultaneously within a single epitaxial device [10,11,12]. This compositional and structural control allows for the broad or multipeak emission necessary for white-light formation without relying on external phosphors or color conversion layers. In addition, achieving high crystal quality in GaN layers during MOCVD growth is crucial, as the evolution of dislocation density, impurity incorporation, and strain management directly impacts the optical and structural properties of the resulting LEDs [18]. Furthermore, the current injection level plays a crucial role in determining the spectral balance of emitted light [19,20,21]. As the current density increases, higher-energy electronic states in the QWs are filled owing to the band-filling effect, leading to a spectral blueshift [22,23,24]. This mechanism enables dynamic tuning of the emission color by simply varying the drive current, offering a flexible route to modulate the balance between long- and short-wavelength components in multiwavelength LEDs [19,20,21]. In this study, we propose a novel and practical approach for achieving current-density-controlled white-light generation using lateral device size scaling. By fabricating LEDs with varying lateral dimensions (from 50 to 800 µm), we induced different current densities across the devices under the same injection current. To enable multi-wavelength emission, the active region consists of a dual QW design: five InGaN QWs tailored for blue emission and a single QW grown near the p-side for long-wavelength orange emission. The lower blue-emitting QWs not only contribute to blue-light generation but also introduce strain, which promotes phase separation in the last QW through spinodal decomposition, leading to a broadened emission between ~500 nm and 580 nm. Larger LEDs exhibit lower current densities, leading to dominant recombination in the upper In-rich QW and long-wavelength emission, whereas smaller LEDs naturally operate at higher current densities, enhancing hole injections into the deeper QWs and promoting blue emission. This structure enables precise modulation of both the CIE 1931 chromaticity coordinates and the correlated color temperature (CCT) of the emitted light, offering a simple and monolithic route to tunable white-light generation.

## 2. Materials and Methods

The InGaN/GaN MQW LEDs used in this study were epitaxially grown on c-plane sapphire substrates via home-made metal–organic chemical vapor deposition (MOCVD). Initially, a 25 nm low-temperature GaN buffer layer was deposited at 550 °C under a reactor pressure of 200 Torr, using trimethylgallium (TMGa) and ammonia (NH_3_) as precursors with a V/III ratio of 5000. The NH_3_ and TMGa flow rates were 3 slm and 10 sccm, respectively. Subsequently, a 2.0 µm thick n-type GaN layer (n_e_ = 2.0 × 10^18^/cm^3^) was grown at 1030 °C under a pressure of 200 Torr with a V/III ratio of 3000. The TMGa and NH_3_ flow rates were adjusted to 50 sccm and 3 slm, respectively, and SiH_4_ was used for n-type doping. The active region consists of six quantum wells with different indium compositions and growth temperatures. The five lower QWs, composed of 2.5 nm thick In_0.15_Ga_0.85_N wells and 10.0 nm thick GaN barriers, were grown at 750 °C with a growth pressure of 300 Torr and were designed for blue emission. The uppermost single QW, composed of In_0.3_Ga_0.7_N/GaN, was grown at 700 °C and is intended to emit in the yellow region. For both InGaN QWs structures, the TMIn and TMGa flow rates were set to 100 sccm and 2.0 sccm, respectively, while maintaining an NH_3_ flow of 5 slm to achieve the desired indium incorporation. The indium composition of the QWs was controlled solely by adjusting the growth temperature without changing the precursor flow rate. This dual-wavelength MQW structure was followed by the deposition of a 100 nm thick p-type GaN layer (n_h_ = 2.0 × 10^17^/cm^3^) at 1000 °C under 200 Torr using Cp_2_Mg as the p-type dopant precursor to complete the LED epitaxial structure. The device fabrication followed standard photolithography and metallization procedures. Ti/Al (20/100 nm) layers were deposited and annealed to form ohmic contacts on n-type GaN, while Ni/Au (10/100 nm) bilayers were deposited on the p-GaN surface as p-type electrodes. Mesa structures defining the LED areas were formed via inductively coupled plasma dry etching using Cl_2_/Ar gas, and device isolation was performed using standard etching techniques. The fabricated LEDs had lateral sizes of 50 × 50, 100 × 100, 200 × 200, 400 × 400, and 800 × 800 µm² and are referred to as 50-LED, 100-LED, 200-LED, 400-LED, and 800-LED, respectively.

For structural and material characterization, high-resolution X-ray diffraction (HRXRD) measurements were performed using a DMAX 2200 (Rigaku Corporation, Tokyo, Japan) to evaluate the periodicity and crystalline quality of the MQW structures. Cross-sectional transmission electron microscopy (TEM) was conducted using a JEM-2100F system (JEOL Ltd., Tokyo, Japan) to investigate the thickness, interface sharpness, and indium distribution in the quantum wells. Electroluminescence (EL) spectra were collected under various injection currents to study wavelength shifts and color evolution. Current–voltage (I–V) and light output–current (L–I) characteristics were measured using a parameter analyzer (HP 4155A, Santa Rosa, CA, USA) and a calibrated photometric system. All measurements were performed at room temperature under a continuous wave operation.

## 3. Results and Discussion

**Figure 1:**
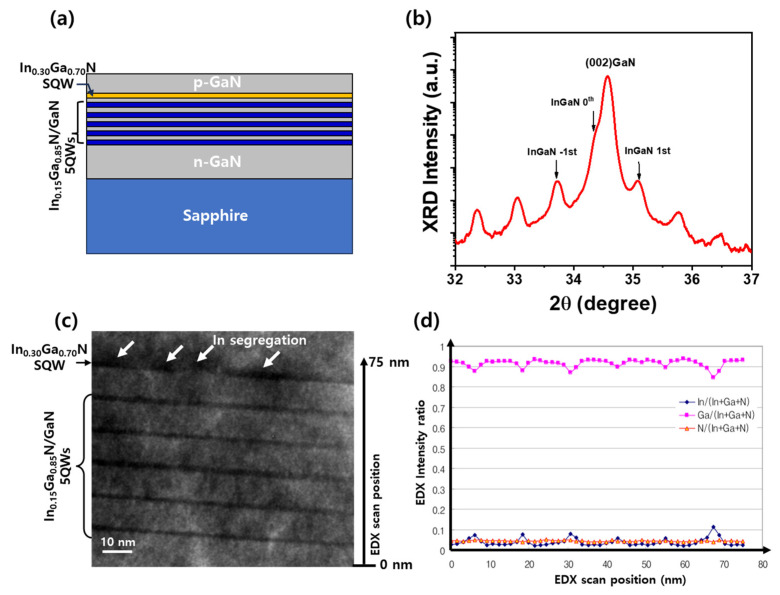
(**a**) Schematic of the GaN-based LED structure with five-period In_0.15_Ga_0.85_N/GaN QWs and a single thin In_0.3_Ga_0.7_N QW grown on sapphire. (**b**) HRXRD ω–2θ scan showing distinct satellite peaks, indicating excellent QW periodicity and interface quality. (**c**) HR-TEM image confirming uniform interfaces in the lower QWs and In segregation in the top QW due to high In content. (**d**) EDX line scan revealing increased In concentration in the upper QW, consistent with the designed dual-composition structure.

Figure 1a illustrates a schematic of a GaN-based LED structure grown on a sapphire substrate. The structure comprises a 2.0 μm thick n-type GaN layer grown on a sapphire substrate, followed by an active region consisting of two distinct types of InGaN/GaN QW structures. The lower section contains five periods of 2.5 nm thick In_0.15_Ga_0.85_N well layers and 10 nm GaN barrier layers, which are designed to emit blue light. In addition, a single QW with a 2.0 nm thick In_0.3_Ga_0.7_N well was embedded as the uppermost active layer, specifically intended to generate yellow emission. This combination of QW structures enables multiwavelength emission within a monolithic device. The entire active region was capped with a 100 nm thick p-GaN layer. Figure 1b shows the high-resolution X-ray diffraction (HRXRD) ω–2θ scan of the InGaN-based LED structure. Distinct satellite peaks higher than the third order are observed around the InGaN 0th peak. Despite the inclusion of QWs with two different In compositions, the clear visibility of high-order satellite peaks indicates excellent periodicity and well-defined interfaces, demonstrating superior interface quality and structural uniformity across multilayer QWs [25]. It is worth noting that the observed diffraction pattern is likely dominated by the lower five-period In_0.15_Ga_0.85_N/GaN QWs structure, as both the lower and upper QWs have comparable thicknesses, and the only difference lies in the indium composition—15% for the five lower QWs and 30% for the single upper QW. Due to the limitations in the resolution of our HR-XRD equipment, the difference in indium composition between these wells did not produce a sufficiently large lattice contrast to be clearly distinguished in the satellite peaks. Consequently, the periodic modulation observed in the XRD scan mainly reflects repetitive lower QWs, and the contribution of the topmost In-rich QW is not readily resolved. To more accurately identify the structural characteristics and confirm the compositional inhomogeneity of indium, additional high-resolution transmission electron microscopy (HR-TEM) analysis was performed. Figure 1c presents an HR-TEM image, confirming the excellent uniformity and sharp interfaces of the lower five-period In_0.15_Ga_0.85_N/GaN QWs structure. These well-defined layers are consistent with the periodicity observed in the HRXRD results. However, the topmost In_0.3_Ga_0.7_N QW layer exhibits a relatively non-uniform indium distribution compared to the intended 2.5 nm design. This inhomogeneity is attributed to the higher In composition, which increases the thermodynamic driving force for phase separation due to the limited miscibility of indium in GaN [10,26,27,28]. In particular, the presence of the underlying blue-emitting In_0.15_Ga_0.85_N QWs also induced additional strain on the topmost QW, further promoting spinodal decomposition. The combination of high In content, ultra-thin well thickness, and strain accumulation promotes spinodal decomposition, leading to indium clustering and compositional fluctuations within the QW [26]. These effects result in local bandgap variations that can broaden the emission spectrum (500–580 nm) and lead to longer-wavelength components, as observed in optical measurements [10]. The contrast difference and blurred interface in the topmost QW further support the presence of In segregation and thickness nonuniformity induced by kinetic limitations during low-temperature growth [29]. 

Figure 1d shows the energy-dispersive X-ray spectroscopy (EDX) line scan profiles of indium (In), gallium (Ga), and nitrogen (N) across the QW region. The elemental intensity ratios clearly confirm the compositional difference between the lower multiple QWs and the uppermost QW. A noticeable increase in the In content was observed at the position of the topmost QW, consistent with the intentional design of a higher indium concentration for yellow emission, which is consistent with the In segregation features observed in the TEM image. However, the absolute values of the In/(In + Ga + N) ratio appear to be lower than those of the nominal 15% and 30% designed compositions. This discrepancy is attributed to the intrinsic limitations of EDX analysis in ultra-thin layers, including the effects of spatial resolution, signal averaging across adjacent barrier layers, and overlap of characteristic X-ray signals. Additionally, partial phase separation in the In_0.3_Ga_0.7_N layer can lead to lateral In inhomogeneity, further reducing the locally averaged In signal detected by the line scan. Despite these limitations, the relative compositional contrast between low- and high-In QWs was clearly resolved, supporting the intended dual-wavelength QW structure. These results verify the successful realization of a compositionally controlled active region, which enables multiwavelength emission capability in the fabricated LED.

**Figure 2:**
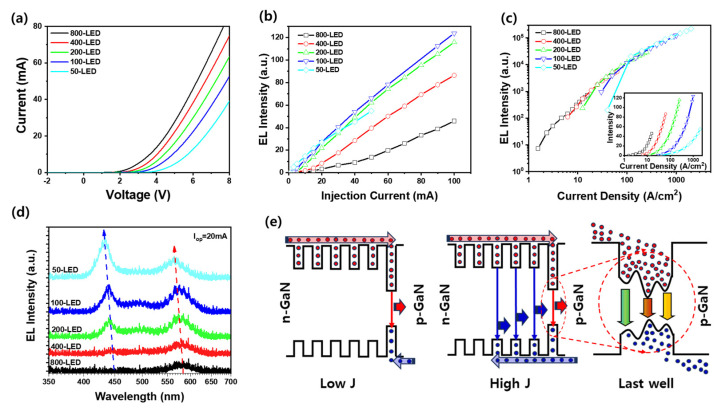
(**a**) Current (I)–voltage (V) and (**b**) light output power (L)–current (I) curves of LEDs with different sizes. (**c**) Light output vs. current density shows consistent trends across all sizes, indicating uniformity; inset shows that larger LEDs yield higher brightness at the same current density. (**d**) EL spectra at 20 mA showing size-dependent blueshift from 580 nm to 445 nm as recombination shifts from the last to lower QWs. (**e**) Emission mechanism: low current favors emission from the last QW; high current enables hole transport to lower QWs. Emission broadening near 580 nm is due to In segregation.

Figure 2a shows the I–V characteristics of LEDs with sizes ranging from 800 µm to 50 µm. As the LED size decreases, the operating voltage at 20 mA increases significantly from 4.42 V (800-LED) to 6.81 V (50-LED). This pronounced increase is primarily attributed to two key factors: increased contact resistance and reduced effective current-spreading area [19]. In smaller LEDs, the contact electrode area is significantly reduced, leading to higher contact resistance due to a limited carrier injection area. Additionally, the reduction in lateral dimensions confines the current flow to a smaller cross-sectional area, thereby increasing the series resistance within the device. Unlike larger LEDs, which benefit from wider lateral current spreading, smaller LEDs experience localized current crowding beneath the contact pad, which not only increases the local resistance but also results in a higher forward voltage. These effects become more prominent as the LED size shrinks, leading to an observed size-dependent increase in the operating voltage [20,22]. Figure 2b presents the light output as a function of the injection current for LEDs of varying lateral sizes. As the LED size decreased from 800 to 100 µm, the optical output steadily increased. This trend mainly results from the higher current density in smaller LEDs, which enhances the carrier injection and improves the radiative recombination in the active region. A higher current density facilitates stronger emission from the InGaN quantum wells, particularly from the higher-energy blue-emitting wells, resulting in greater light output. However, when the LED size was further reduced to 50 µm, a deviation from this trend was observed, that is, a reduction in the optical output at the same current level. This decrease is mainly attributed to the combined effects of the increased series and contact resistance, which results in a higher operating voltage and elevated power consumption [22]. The higher resistance also limits the efficient current spreading and leads to severe current crowding in the contact region. These factors intensify local Joule heating, even at moderate current levels, which can degrade internal quantum efficiency due to the increased non-radiative recombination pathways. Figure 2c presents the light output of LEDs with different sizes as a function of the current density. When normalized for the LED area, all LEDs exhibited nearly identical trends regardless of the LED size, demonstrating that the light output increased linearly with the current density across all LEDs. This consistent behavior strongly reflects the high degree of process uniformity and reproducibility achieved across a single wafer, confirming that the differences in EL intensity shown in Figure 2b are primarily due to size scaling rather than material or fabrication variations. The inset of Figure 2c provides additional insight by showing that, under the same current density, the 800-LED produces a higher absolute light output than smaller devices. Unlike Figure 2b, where the same current is applied, the inset compares LEDs under equivalent current density conditions, isolating size-dependent effects. The superior performance of the larger LED at the same current density is attributed to its lower operating voltage, which reduces electrical power loss and improves the overall wall-plug efficiency. Moreover, larger LEDs benefit from better thermal management due to their greater surface area, which minimizes the junction temperature rise during operation. This thermal stability suppresses non-radiative recombination processes, maintaining a higher radiative recombination efficiency and, thus, better light output. In contrast, smaller LEDs suffer from increased series resistance and voltage drop, along with higher localized heating due to limited heat dissipation. The elevated junction temperature in smaller devices enhances non-radiative recombination pathways, such as defect-related recombination and Auger recombination, leading to efficiency degradation, even at the same current density. It should be emphasized that because all devices were fabricated from a single epitaxial wafer without any variation in growth conditions, differences in threading dislocation density can be reasonably excluded as a factor influencing the observed EL emission trends. These results highlight that although normalized output trends are uniform, larger LEDs retain an advantage in absolute light emission owing to better electrical characteristics under identical current density conditions. 

Figure 2d shows the EL spectra at 20 mA for LEDs with different lateral sizes. In the largest 800-LED, the dominant emission appears at approximately 580 nm, indicating that radiative recombination primarily occurs in the last QW composed of In_0.3_Ga_0.7_N. This is attributed to the intrinsic properties of III-nitride semiconductors, where electrons have a higher concentration and significantly higher mobility than holes [30,31,32]. As a result, under a low current density, electrons are more efficiently transported and accumulated near the p-type layer, while holes, owing to their limited mobility and diffusion length, tend to recombine in the QW closest to the p-contact, in this case, the last QW. However, as the LED size decreases, and thus the current density increases under the same bias current, the increased hole injection facilitates the deeper penetration of holes into the active region. This allows the recombination to gradually shift from the upper In_0.3_Ga_0.7_N QW toward the lower In_0.15_Ga_0.85_N QWs. Consequently, the emission peak shifts from 580 nm to shorter wavelengths, such as 500 nm and eventually to 450 nm, reflecting transitions in lower-bandgap QWs. This spectral blueshift is not only influenced by band-filling effects, but also by improved carrier distribution across multiple QWs owing to stronger hole injection at high current densities [24,30]. Figure 2e schematically illustrates the emission mechanism. At low current densities, recombination is dominated by the last QW near the p-side, owing to its proximity and strong carrier localization. At higher current densities, enhanced hole transport allows radiative recombination to occur in both the upper and lower blue-emitting QWs. To further support this interpretation, carrier distribution behaviors at low and high current densities within III-nitride quantum wells have been reported in the literature [31], showing that electrons predominantly accumulate near the p-side QWs at low injection levels, whereas enhanced hole injection at higher currents leads to a more uniform carrier distribution across multiple QWs. Moreover, the broadened emission at approximately 500–580 nm from the last QW is attributed to In segregation and spinodal decomposition [26,27], which create compositional inhomogeneities, as shown in Figure 1c, resulting in a wide spectral range of yellow to green emission.

**Figure 3:**
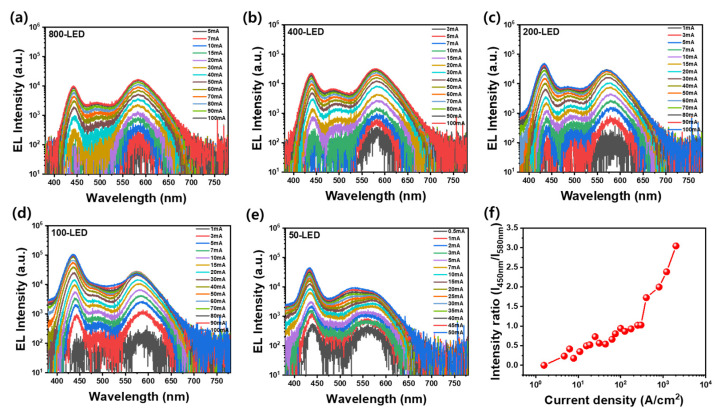
EL spectra of LEDs with different sizes: (**a**) 800 µm, (**b**) 400 µm, (**c**) 200 µm, (**d**) 100 µm, and (**e**) 50 µm, under increasing injection current. As size decreases, blue emission (~450 nm) becomes dominant over orange (~580 nm) due to higher current density and band-filling effects. (**f**) Intensity ratio (I_450nm_/I_580nm_) as a function of the current density shows enhanced blue emission in smaller devices.

Figure 3a–e present the EL spectra of LEDs with various lateral sizes (800 µm to 50 µm) under increasing injection currents, highlighting how the current density influences the carrier distribution and emission wavelength in the QW structure. In GaN-based LEDs, because of the intrinsic disparity in carrier properties, namely the higher concentration and mobility of electrons compared to holes, radiative recombination at low current densities tends to occur preferentially in the last (topmost) In_0.3_Ga_0.7_N QW located near the p-side [30,32]. This exhibits strong carrier localization owing to its high In content and structural non-uniformity, leading to dominant emission around 580 nm in large LEDs like the 800-LED. 

As current density increases, either by increasing the injection current or by reducing the LED size, hole injections and transport become more effective. This allows holes to reach deeper QWs farther from the p-contact, especially lower In_0.15_Ga_0.85_ N QWs with a higher bandgap. Consequently, the emission spectrum shifts from red to blue, reflecting recombination in progressively shallower QWs. This trend is clearly observed in the 50-LED (Figure 3e), which displays a strong blue emission near 450 nm, dominated by recombination in the first to fifth QWs. The intermediate emission around 500 nm becomes more apparent in mid-sized devices (e.g., 200-LED) and is attributed to a partial In phase separation in the last QW. This spectral evolution is further quantified in Figure 3f, where the intensity ratio (I_450nm_/I_580nm_) of the blue to red emission is plotted against the current density for all LED sizes. The intensity ratio increases with the current density, particularly in smaller LEDs, indicating a shift in the dominant emission wavelength from orange (580 nm) to blue (450 nm). This reflects both the onset of band-filling effects and enhanced hole diffusion into lower QWs under high-injection conditions [24,32]. The results demonstrate that the overall color of light emitted from the LED can be precisely tuned by controlling the current density, which is inherently linked to the LED size and bias conditions. These findings underscore the importance of QW design and injection control in enabling tunable, multiwavelength LED emission.

**Figure 4:**
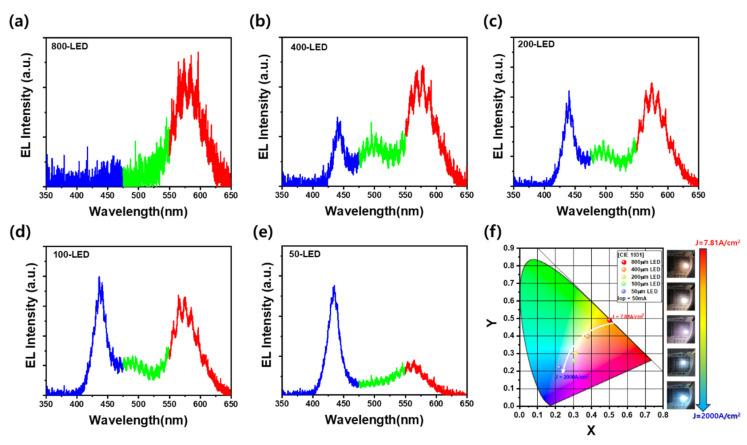
EL spectra of LEDs with sizes of (**a**) 800 µm, (**b**) 400 µm, (**c**) 200 µm, (**d**) 100 µm, and (**e**) 50 µm under 50 mA injection. As size decreases, emission shifts from 580 nm (orange) to 450 nm (blue) due to higher current density and deeper hole injection. (**f**) CIE 1931 color coordinates showing color transition from orange to sky-blue with decreasing LED size.

Figure 4a–e show the EL spectra of LEDs with varying lateral sizes—800 µm, 400 µm, 200 µm, 100 µm, and 50 µm—under a constant injection current of 50 mA. Although the same current is applied, the effective current density increases significantly as the LED area decreases. In the 800-LED, the EL spectrum is dominated by a peak around 580 nm, corresponding to orange-yellow emission from the In-rich last QW. As the LED size decreases to 400 µm, additional emission components near 500 and 450 nm emerge, although the 580 nm peak remains dominant. This indicates that recombination still primarily occurs in the last QW, but hole injection to deeper wells is becoming more active. In the 200-LED (Figure 4c), the intensity of the 450 nm blue emission becomes comparable to that of the 580 nm peak, suggesting balanced recombination in both the top and lower QWs. Correspondingly, the calculated color rendering index (CRI) increases significantly for intermediate-sized LEDs: the 200-LED and 400-LED exhibit high CRI values of approximately 83 and 85, respectively, due to their balanced multi-wavelength emissions, while the 800-LED and 50-LED show lower CRI values near 68 and 35, respectively, because of narrowband emissions. As the LED size decreases to 100 µm and 50 µm, the EL spectra are increasingly dominated by the 450 nm peak, indicating that recombination shifts toward the lower five In_0.15_Ga_0.85_N/GaN QWs. This behavior is consistent with the carrier dynamics in III-nitride semiconductors, where electrons have higher mobility and concentration than holes [30,32]. It is also noteworthy that the spectral full width at half maximum (FWHM) varies depending on the emission region: the blue peak exhibits a narrow FWHM of 17.8 nm, whereas the green and orange emissions show broader FWHMs of 58 nm and 46.2 nm, respectively. This broadening is attributed to indium compositional inhomogeneity and spinodal decomposition occurring in the topmost In_0.3_Ga_0.7_N QW. Among these, the green emission not only shows the largest FWHM but also presents the lowest emission intensity, reflecting greater compositional fluctuation and weaker radiative recombination efficiency. As the current density increases in smaller LEDs, hole injections become more effective, allowing carriers to reach deeper QWs and favoring recombination in higher-bandgap regions. Figure 4f plots the CIE 1931 chromaticity coordinates extracted from the EL spectra of LEDs with different sizes at 50 mA. As indicated by the white arrow, the color coordinates shift continuously from orange (0.501, 0.490) in the 800-LED to sky-blue (0.224, 0.218) in the 50-LED, passing through white emission (0.379, 0.397) around the 200-LED. This transition clearly demonstrates the feasibility of color tuning through lateral device scaling at a fixed current, enabled by size-dependent carrier distribution and multi-wavelength QW design.

**Figure 5:**
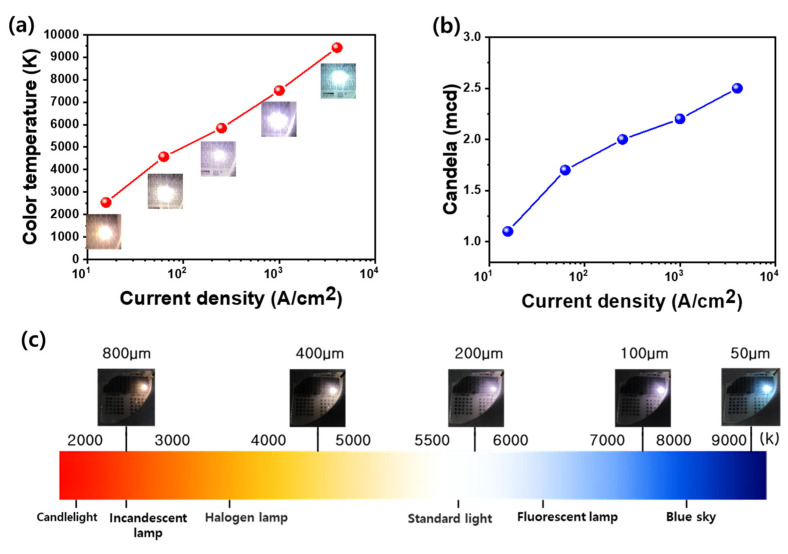
(**a**) Correlated color temperature (CCT) variation of LEDs under different current densities. CCT is tuned from 2536 K to 9425 K by adjusting the current density, independent of LED size. Warm white (2536 K) is achieved at 15.6 A/cm^2^ (800-LED), daylight white (5844 K) at 250 A/cm^2^ (200-LED), and sky-blue (9425 K) at 4000 A/cm^2^ (50-LED). (**b**) Luminous intensity increases from 1.1 mcd to 2.5 mcd with the current density, indicating higher photon output at stronger injection. (**c**) Comparison of LED color temperatures with those of standard light sources, showing the transition from halogen-like warm white to cool daylight and beyond.

Figure 5 highlights the correlation between current density and emission properties of LEDs with varying sizes, focusing on color temperature and luminous intensity. In Figure 5a, the correlated color temperature (CCT) is plotted as a function of injection current density. As the current density increases—mainly due to reduced LED size—the CCT of the emitted light significantly increases. Larger 800-LEDs operating at lower current densities exhibit warm-white emission with lower CCT values (~2500 K), similar to halogen lamps. In contrast, smaller LEDs, such as the 50-LED, emit a much colder white light with a CCT exceeding 9000 K, indicating a shift toward bluish-white emission. This trend aligns with the previously discussed band-filling effect, where high current densities enhance carrier recombination in higher-energy QWs, favoring shorter-wavelength (blue) emission. Notably, the achieved CCT tunability from approximately 2500 K to over 9000 K is comparable to or even broader than that reported for conventional phosphor-converted white LEDs (typically 2700–6500 K) and recent phosphor-free multi-quantum-well LEDs (typically 4000–8000 K), highlighting the wide spectral control enabled by our size-scaling method [6,33,34]. Figure 5b shows the change in luminous intensity (in millicandela, mcd) as a function of the current density. The light output increases with the current density up to a certain point, but smaller LEDs show signs of optical saturation or thermal quenching at very high current densities. While the absolute light output is higher in larger LEDs due to greater emission area, smaller LEDs offer higher brightness per unit area, which can be beneficial for high-resolution or miniaturized lighting applications. Figure 5c provides visual examples of white-light emission across different LED sizes, annotated with the corresponding CCT values. The images range from warm yellowish-white (lower CCT) to bright bluish-white (higher CCT), illustrating size-tunable white color generation. This demonstrates that white light with different color temperatures can be achieved without changing the material structure, simply by adjusting the LED size and, hence, the current density. These results underline the feasibility of the current density engineering as a powerful method for controlling white-light characteristics in compact LED platforms.

## 4. Conclusions

We demonstrated tunable white-light emission from a monolithic GaN-based LED wafer by varying the lateral size of individual LEDs, thereby modulating the current density and carrier distribution. At a fixed injection current, larger devices (e.g., 800-LED) exhibit dominant orange emission near 580 nm with a CIE coordinate of (0.501, 0.490) and a correlated color temperature (CCT) of 2536 K, while smaller devices (e.g., 50-LED) show strong blue emission at 450 nm with a CIE coordinate of (0.224, 0.218) and a CCT of 9425 K. This shift is attributed to the disparity in electron and hole concentrations and mobilities in III-nitride semiconductors. Under the low current density, recombination primarily occurs in the topmost In_0.3_Ga_0.7_N QW, which is located near the p-GaN and exhibits phase-separated indium clusters due to spinodal decomposition driven by high In content and thin well thickness. As the current density increases, hole injections improve, allowing recombination to shift into lower In_0.15_Ga_0.85_N QWs, resulting in blue-shifted emission. The ability to achieve color tuning from warm white to sky-blue across a single wafer through device scaling offers a simple yet powerful strategy for integrated white LED applications without relying on phosphors or multiple epitaxial structures.

## Data Availability

The data presented in this study are available on request from the corresponding author. The data are not publicly available due to privacy concerns.
